# Manipulation of fatty acid profile and nutritional quality of *Chlorella vulgaris* by supplementing with citrus peel fatty acid

**DOI:** 10.1038/s41598-022-12309-y

**Published:** 2022-05-17

**Authors:** Kourosh Ghodrat Jahromi, Zhila Heydari Koochi, Gholamreza Kavoosi, Alireza Shahsavar

**Affiliations:** 1 Department of Biotechnology, School of Agriculture, Shiraz University, Shiraz, Islamic Republic of Iran; 2 Department of Horticultural Science, School of Agriculture, Shiraz University, Shiraz, Islamic Republic of Iran

**Keywords:** Biochemistry, Biological techniques

## Abstract

Microalgae could be an excellent resource of functional and essential fatty acids. To achieve viable microalgal biomass production, mass cultivation of microalgae is required; however, the high cost of nutrients is the obstacle. An inexpensive and nutritious material is required to feed *Chlorella vulgaris* in the pharmaceutical and food sectors. Citrus peel waste with a valuable nutritional quality could be one of the promising and inexpensive candidates. In this study, the fatty acid extract from different citrus peels was used as the organic nutrient source for the cultivation of *Chlorella*. The proximate composition of bitter orange, sweet orange, grapefruit, and mandarin peels were determined, and their nutritional quality was evaluated. Total fatty acids from the citrus peel were prepared by acidic methanol hydrolysis and hexane extraction. Fourier transforms infrared (FT-IR) and gas chromatography–mass spectrometry (GC–MS) was used to analyze the fatty acid composition and nutrient composition. Fatty acids from the citrus peels were added to the *Chlorella* culture medium to study their influences on biomass, lipid production, fatty acid profile, and nutritional quality of *Chlorella*. The most predominant citrus peel fatty acids were linoleic, palmitic, oleic, linolenic, and stearic acids. The citrus peels contain polyunsaturated, saturated, and monounsaturated fatty acids. The most unsaturated fatty acids were omega-6, omega-3, omega-9, and omega-7. The citrus peel had acceptable atherogenicity, thrombogenicity, omega-6/omega-3, peroxidizability, hypocholesterolemic, and nutritive value indices. The major fatty acids of *Chlorella* were palmitic, linoleic, oleic, alpha-linolenic, gamma-linolenic, 4,7,10,13-hexadecatetraenoic, palmitoleic, 7,10-hexadecadienoic, 7,10,13-hexadecatrienoic, lauric and 5,8,11,14,17-eicosapentaenoic acids. *Chlorella* contains polyunsaturated, saturated, and monounsaturated fatty acids. The most unsaturated fatty acids contain omega-6, omega-3, omega-9, and omega-7. *Chlorella* had acceptable atherogenicity, thrombogenicity, omega-6/omega-3, hypocholesterolemic, peroxidizability, and nutritive value indices. Supplementation of *Chlorella* with citrus peels fatty acid increases total biomass, lipid content, and nutritional quality of *Chlorella*. The present research shows that citrus peels have good nutritional quality and could be used for the inexpensive cultivation of *Chlorella* biomass with potential utility for food application.

## Introduction

Citrus is one of the most extensively grown horticultural fruits. The sweet orange, bitter orange, lemon, lime, grapefruit, and mandarin are the most extensively farmed and industrially important citrus fruits globally in the agro-industrial area. Citrus fruit may be used to make fresh juice, citrus-based drinks, and jelly, in addition to being consumed. Juice for beverages, jellies, marmalades, potpourris, jams, candied peel, flavoring mediator for beverages, oils and essences, fiber, and pectin for food components are the chief goods of the citrus processing industry^1^. Citrus fruit is mostly made up of pulp (71%), peel (27%), and seeds (2.0%). Pectin (21%), citric acid (16%), pentosans (15%), fiber (11%), glucoside (10%), minerals (10%), proteins (9.0%), essential oils (5.0%), and lipids (3.0%) make up the dry matter^2^. The derivative of the citrus processing industry is the citrus peel waste that accounts for 50–60% of the citrus processed, depending on the citrus cultivar. Peels (50%), internal tissue (30%), and seeds (10%) make up citrus peel waste with non-nutrient components like essential oil and nutrients like amino acids and fatty acids^3^. Citrus peel waste is now utilized primarily as animal feedstuff, an organic soil conditioner, a composting substrate, and bioethanol and biomethanol production substrate. Citrus peel waste has a high added value and may be used to make pectin, dietary fibers, protein, and fatty acids in the food sector. Citrus peel waste might potentially be used to extract flavonoids, favorable agents, and citric acid in the cosmetic and pharmaceutical sectors^4^. The macronutrient composition and profile of fatty acids, on the other hand, have been investigated to a lesser extent. Citrus peel waste offers enough nutrients for microalgae culture to enhance lipid and fatty acid content in the microalgae.

Microalgae are suitable for the food industry due to their quick growth rate and are used as a primary source of lipid and protein^5^. Proteins, lipids, carbohydrates, vitamins, pigments, and carotenoids are all in microalgae, the potential resource of biological constituents^6^. Microalgae lipid have also been shown to be harmless for digestion in numerous human and animal investigations, and it has been linked to favorable health outcomes such as decreased blood glucose, blood pressure, and cholesterol^7^. Microalgae are an excellent resource of proteins, and essential and non-essential amino acids, lipids, sugars, essential fatty acids, vitamin precursors, and mineral deposits, as well as organic acid, terpenoids, alkaloids, steroids, and phenolic compounds, all of which have been used as therapeutic foods to prevent hypertension, hypercholesterolemia, atherosclerosis, and diabetes mellitus^8^. Various studies reported that salinity and light intensity could improve lipid accumulation and manipulate fatty acid profiles in the microalgae^9^. Besides the light intensity, a nutritious and affordable substance for microalgae feeding is required to employ *Chlorella* in the food and pharmacological industries. These objectives can be met by feeding microalgae with nutritive ingredients and promoting fatty acids^10^. An inexpensive and nutritious material is required to feed *Chlorella* in the pharmaceutical and food sectors and manufacture biofuels to encourage the synthesis of lipids and fatty acids for this purpose^11^. Citrus peel waste with a valuable nutritional quality could be one of the promising and inexpensive candidates. But the direct use of citrus peel has several limitations to microalgae culture due to antinutrient materials in the citrus peels. Partial extraction of fatty acid reduces antinutrients content and overcomes this problem^12^.

Accordingly, in this research, the fatty acid extract from different citrus peels was used as the organic nutrient source for cultivating *Chlorella*. The nutritional quality of extracted fatty acids from bitter orange (*Citrus aurantium*), sweet orange (*Citrus sinensis*), grapefruit (*Citrus paradisi*), and mandarin (*Citrus reticulata*) peel waste and the stimulatory effects of their fatty acids on the manipulation of fatty acid composition and nutritional quality of *Chlorella* are presented. The macronutrient composition, fatty acid profile, and lipid nutrition quality of citrus peel waste and *Chlorella* supplemented with fatty acid from citrus peel have been explored in this research for the first time so far.

## Materials and methods

The bitter orange, sweet orange, grapefruit, and mandarin were grown at Fars Research Center for Agriculture and Natural Resources (Jahrom, Fars, Iran). Fresh peels from ripped, harvested fruits were got from randomly chosen, healthy trees in December. Jahrom Agricultural and Natural Resources Research Station are allocated at an altitude of 1070 m with the longitude of 53°, 37ʹ, 33ʺ, and a latitude of 28°, 30ʹ, 48ʺ. Jahrom Agricultural and Natural Resources Research Station confirmed all the source and batch number details. A voucher specimen has been deposited at the Herbarium of the Agricultural and Natural Resources Research Station. The characteristics of citrus trees in this research station were recorded in the book of Citrus cultivars of Jahrom Agricultural Research Station^13^. Experimental research and field studies on plants, including the collection of plant material, comply with relevant institutional, national, and international guidelines and legislation.

The peel was removed from the fruit and dried at room temperature before being crushed in a home grinder. The standardized protocols proposed in the literature were used to evaluate moisture content, ash content, total carbohydrate, total protein, total lipid, protein digestibility, and total energy of microalgae by following the Association of Official Analytical standard procedures Chemists method^14^. Fourier transforms infrared (FT-IR) spectroscopy in the range of 4000–400 cm^−1^, done with a Bruker FTIR spectrophotometer (Germany), was used to characterize the chemical components of citrus peels and *Chlorella* supplemented with citrus peel.

### Fatty acids preparation and profiling

Lipids from citrus peel powder (1.0 g) were hydrolyzed and esterified with 5.0 ml of acidic methanol (methanol: sulfuric acid 80:20, v/v) in a shaking water bath for 120 min at 80 °C. At that time, 4.0 mL of normal saline was added to the tubes and vortexed for 5.0 min. Then, 4.0 mL of hexane was added to the tubes and vortexed for 5.0 min, and centrifuged at 3000*g* for 20 min in the next step. The upper hexane phase was extracted and transferred to gas chromatography vials for fatty acid methyl ester analysis. GC–MS analysis was performed using Agilent (Agilent 7890B GC 7955A MSD) device equipped with a silica capillary column (HP-5MS (30 m × 0.25 mm id; thickness 0.25 µm)), coupled with a quadrupole mass spectrometer according to the standardized method that is described elsewhere in detail^15^. For the preparation of free fatty acid, we used acid hydrolysis (without methanol) with the same procedure, followed by the addition of normal saline for better extraction of fatty acid and then hexane extraction. After hexane evaporation, the free fatty acid extract was added to the microalgae culture medium.

### Lipid nutritional quality index

Total saturated fatty acids (SFA), unsaturated fatty acids (UFA), polyunsaturated fatty acids (PUFA), monounsaturated fatty acids (MUFA), omega-6/omega-3 ratio, PUFA/SFA (P/S), hypocholesterolemic index (HI), atherogenicity index (AI), thrombogenicity index (TI), peroxidizability index (PI), and nutritive value index (NVI) were calculated according to the formula described in the literature^16^.

### Microalgal culture and treatment

*Chlorella vulgaris* (IBRC: M50026) was obtained from the Iranian Biological Resources Center (IBRC) (Tehran, Iran). *Chlorella* was isolated using the agar plate procedure, then purified in Bold's Basal Media (BBM) before being transferred to the same liquid medium. The *Chlorella* cells were transferred to BBM liquid medium, bubbled, and maintained at 28 °C with an initial pH of 6.8 and 2500 E m^−2^ s^−1^ light intensity to raise biomass. A mechanical pump aerated the culture after it passed through a 0.22 m filter. The microalgal cells were supplied with fatty acid (5.0 g/1000 ml) at the logarithmic phase (after 12 days), and the cultures were incubated for 4 days^11^. The biomasses from supplemented microalgal cultures were collected by centrifugation at the end of the stationary phase (after 17–18 days). It was washed with deionized water and lyophilized to remove the associated residues. The dry biomass was used to analysis of macronutrients and fatty acid composition^14^. The standardized protocols proposed in the literature were used to extract and profile fatty acids^15^.

### Statistical analysis

The results are presented as averages with standard deviations from three replicates. Through SPSS software version 16, experimental data were evaluated as a completely randomized design using one-way analysis of variance (ANOVA) followed by Tukey post-hoc testing (P ≤ 5%). (SPSS Inc., Chicago, IL, USA). To investigate the apparent difference in the distribution of the components between the samples, the matrix of fatty acid composition and nutritional value data in the samples was evaluated for principal component analysis (PCA) using Minitab software (version 20.1.2).

### Ethics approval

There were no human subjects or animal experiments in our study.

## Results and discussions

### Citrus peel proximate analysis

**Figure 1:**
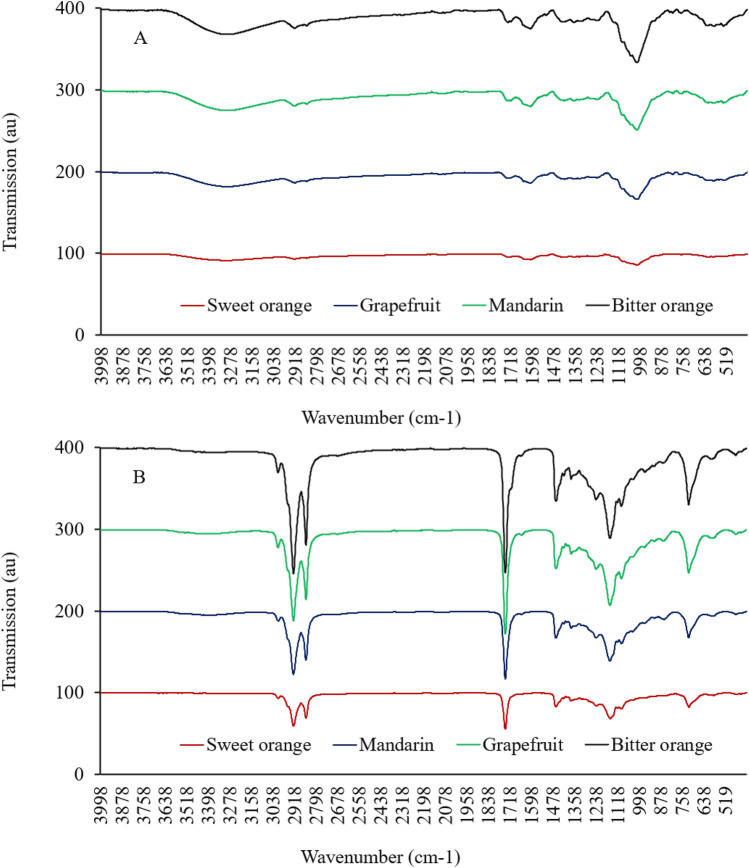
Fourier transform infrared spectrum of peel powder (**A**) and fatty acid (**B**) from bitter orange, sweet orange, grapefruit, and mandarin peels.

The typical FTIR spectrum of bitter orange, sweet orange, grapefruit, or mandarin peels is shown in Fig. 1. The 3700–3100 cm^−1^ bands are associated with stretching vibrations of OH groups in water or hydrogen-bonded OH. Peaks in the 2900–2700 cm^−1^ region are associated with the stretching vibrations of lipid and fatty acid CH, CH2, and CH3 groups. The stretching of C=O amides, C=C aromatics, N–H amines, or carboxyl groups in proteins is represented by the bands in the 1300–1100 cm^−1^ region. Polysaccharide vibrations, including symmetric stretching of C–O–C and OH groups, cause the band at 1100–900 cm^−1^. The bands at 900–500 cm^−1^ are due to the vibration of terpenes and terpenoids^17^. According to FTIR analyses, citrus peels have a high concentration of carbohydrates (1100–900 cm^−1^) and a lower concentration of protein, fatty acids, and terpenes (Fig. 1).

The macronutrient analysis also confirmed that the tested citrus peels have a large carbohydrate and lesser protein, lipid, and fatty acid (Table 1). The macronutrient composition of bitter orange, sweet orange, grapefruit, and mandarin is similar, although there are statistically significant differences (P ≤ 5%). Carbohydrates are the major macronutrient in citrus peels that make them to have high energy value, but the most abundant carbohydrates are fibers (pectin) that cannot be digested^18^. Bitter orange and grapefruit have the similar dry matter, carbohydrate, and fat content, while sweet orange and mandarin consider protein, moisture, ash contents, and digestibility (Table 1 and Fig. S1 in the supplemental file).

**Table 1:** Biochemical composition of bitter orange, sweet orange, grapefruit, and mandarin peels.

Parameters	Bitter orange	Grapefruit	Sweet orange	Mandarin
Moisture (g/100 g fw)	77.50 ± 3.01^a^	75.40 ± 2.93^a^	78.52 ± 3.05^a^	80.32 ± 3.12^a^
Dry matter (g/100 g fw)	22.50 ± 0.87^b^	24.60 ± 0.96^a^	21.48 ± 0.84^b^	19.68 ± 0.77^c^
Ash (g/100 g dm)	8.54 ± 0.33^a^	9.56 ± 0.37^a^	10.85 ± 0.42^a^	9.63 ± 0.37^a^
Carbohydrate (g/100 g dm)	57.42 ± 2.23^a^	58.83 ± 2.29^a^	56.10 ± 2.18^a^	57.85 ± 2.25^a^
Protein (g/100 g dm)	18.50 ± 0.72^a^	17.61 ± 0.68^a^	18.99 ± 0.74^a^	19.20 ± 0.75^a^
Fat (g/100 g dm)	13.83 ± 0.54^a^	12.89 ± 0.50^a^	12.67 ± 0.49^a^	11.56 ± 0.45^a^
Energy (kcal/100 g)	428.15 ± 16.65^a^	421.78 ± 16.40^a^	414.39 ± 16.11^a^	412.24 ± 16.03^a^
Digestibility (g/100 g)	53.70 ± 2.09^a^	52.60 ± 2.05^a^	54.33 ± 2.11^a^	54..25 ± 2.11^a^

The values are expressed as means ± SD for three replicates experiments. Mean values with similar letters within a row are statistically similar, while mean values with different letters within a row are significantly different by the Tukey test (*P* < 0.05).

*fw* fresh weight, *Dm* dry matter.

*Citrus maxima*^19^ and *citrus natsudaidai* ^20^ peels have chemical compositions similar to our findings. Citrus peel waste contains polysaccharides (cellulose and pectin), monosaccharides (sucrose, fructose, glucose, galactose), organic acid (citric, succinic, malic, tartaric, oxalic), fatty acid (palmitic, oleic, linoleic, linolenic, and stearic), and minerals (nitrogen, calcium, magnesium, potassium)^21^. Citrus peel waste also contains volatile constituents (alcohols, ester, aldehydes, ketone, hydrocarbon), flavonoids (flavanones, anthocyanins, flavones), essential oil (limonene and linalool), limonoids (limonin), enzymes (pectin esterase, peroxidase, phosphatase), carotenoids (carotene, lutein), polyphenolic constituents, nitrogen compounds (nitrate, ammonia), and vitamin (B groups vitamins, ascorbic acid)^22^. All of them make citrus peels, the raw and inexpensive materials for foodstuff, ingredients, and flavoring.

### The fatty acid profile of the citrus peel

The typical FTIR patterns of fatty acid extracts of bitter orange, sweet orange, grapefruit, and mandarin peels are shown in Fig. 1. The strong peaks in the 2900–2700 cm^−1^ region are associated with the stretching vibrations of lipid and fatty acid CH, CH2, and CH3 groups. The C=C stretching of unsaturated fatty acids and C=O of carboxyl groups can be related to the variations in FTIR patterns observed in the 1900–1500 cm^−1^ region. The weak bands in the 1200–1000 cm^−1^ might be related to lipophilic volatile hydrocarbon and terpenes^17^. There were no proteins and carbohydrates in prepared extracts of fatty acids.

The fatty acid composition of bitter orange, sweet orange, grapefruit, and mandarin peels are stated in Table 2. The most predominant components in the citrus oil were linoleic acid, palmitic acid, oleic acid, alpha-linolenic acid, stearic acid, palmitoleic acid, and myristic acid. The fatty acid composition of bitter orange, sweet orange, grapefruit, and mandarin remains similar, as there are some statistically significant differences, especially in the oleic acid and alpha-linolenic acid content (P ≤ 5%). Grapefruit had the highest oleic acid and alpha-linolenic acid and sweet orange the lowest, while mandarin had significantly the lowest oleic acid compared to all other citrus peels (P ≤ 5%). The contents of arachidic acid, heptadecanoic acid, docosadienoic acid, palmitic acid, tridecanoic acid, stearic acid, myristic acid, decanoic acid, and pentadecanoic acid were similar in sweet orange and mandarin (P ≤ 5%). At the same time, bitter orange and grapefruit peel fatty acid compositions were similar considering percentages of oleic acid, hexadecadienoic acid (C16:2n−6), gamma-linolenic acid, palmitoleic acid, and dodecanoic acid (Table 2 and Fig. S2 in the supplemental file).

**Table 2:** Fatty acid composition (percent) and lipid nutritional quality of bitter orange, sweet orange, grapefruit, and mandarin peels.

Fatty acid	Bitter orange	Grapefruit	Sweet orange	Mandarin
Decanoic acid (C10:0)	0.44 ± 0.02^b^	0.00 ± 0.00^b^	0.00 ± 0.00^b^	1.59 ± 0.09^a^
Dodecanoic acid (C12:0)	0.00 ± 0.00^b^	0.85 ± 0.05^a^	1.03 ± 0.06^a^	0.00 ± 0.00^b^
Tridecanoic acid (C13:0)	0.00 ± 0.00^b^	0.00 ± 0.00^b^	0.60 ± 0.03^a^	0.56 ± 0.03^a^
Tetradecanoic acid (C14:0)	0.75 ± 0.04^b^	1.04 ± 0.06^b^	1.65 ± 0.09^a^	2.26 ± 0.13^a^
Pentadecanoic acid (C15:0)	0.00 ± 0.00^c^	0.35 ± 0.02^b^	0.50 ± 0.03^a^	0.51 ± 0.03^a^
Hexadecanoic acid (C16:0)	27.97 ± 1.59^a^	22.52 ± 1.28^b^	29.38 ± 1.67^a^	28.97 ± 1.64^a^
9-Hexadecenoic acid (C16:1n7)	2.50 ± 0.14^a^	2.12 ± 0.12^a^	1.62 ± 0.09^b^	1.97 ± 0.11^a^
7,10-Hexadecadienoic acid (C16:2n6)	0.00 ± 0.00^b^	0.73 ± 0.04^a^	0.00 ± 0.00^b^	0.00 ± 0.00^b^
Heptadecanoic acid (C17:0)	0.65 ± 0.04^b^	0.43 ± 0.02^c^	0.72 ± 0.04^b^	1.06 ± 0.06^a^
Octadecanoic acid (C18:0)	4.46 ± 0.25^a^	2.95 ± 0.17^c^	3.82 ± 0.22^b^	4.70 ± 0.27^a^
9-Octadecenoic acid (C18:1n9)	19.86 ± 1.13^a^	20.80 ± 1.18^a^	17.13 ± 1.01^a^	9.76 ± 0.55^b^
9,12-Octadecadienoic acid (C18:2n6)	33.20 ± 1.88^a^	30.30 ± 1.72^a^	33.90 ± 1.92^a^	30.08 ± 1.71^a^
9,12,15-Octadecatrienoic acid (C18:3n3)	8.20 ± 0.46^b^	16.10 ± 0.91^a^	6.35 ± 0.36^c^	15.40 ± 0.87^a^
6,9,12-Octadecatrienoic acid (C18:3n6)	0.00 ± 0.00^b^	0.36 ± 0.02^a^	0.00 ± 0.00^b^	0.00 ± 0.00^b^
6,9,12,15-Octadecatetraenoic acid (C18:4n3)	0.00 ± 0.00^a^	0.00 ± 0.00^a^	0.00 ± 0.00^a^	0.00 ± 0.00^a^
Eicosanoic acid (C20:0)	1.32 ± 0.07^a^	0.75 ± 0.04^b^	1.57 ± 0.09^a^	1.77 ± 0.10^a^
Arachidonic acid (C20:0)	0.00 ± 0.00^a^	0.00 ± 0.00^a^	0.00 ± 0.00^a^	0.00 ± 0.00^a^
5,8,11,14,17-Eicosapentaenoic acid (C20:5n3)	0.00 ± 0.00^a^	0.00 ± 0.00^a^	0.00 ± 0.00^a^	0.00 ± 0.00^a^
13,16-Docosadienoic acid (C22:2n6)	0.54 ± 0.03^b^	0.36 ± 0.02^b^	0.90 ± 0.05^a^	0.92 ± 0.05^a^
Docosahexaenoic acid (C22:6n3)	0.00 ± 0.00^a^	0.00 ± 0.00^a^	0.00 ± 0.00^a^	0.00 ± 0.00^a^
Total	99.89 ± 5.66^a^	99.66 ± 5.65^a^	99.77 ± 5.66^a^	99.55 ± 5.64^a^
Saturated fatty acid (SFA)	35.59 ± 2.02^b^	28.89 ± 1.64^c^	39.27 ± 2.23^a^	41.42 ± 2.35^a^
Unsaturated fatty acid (UFA)	64.30 ± 3.65^ab^	70.77 ± 4.01^a^	60.50 ± 3.43^b^	58.13 ± 3.30^b^
UFA/SFA	1.81 ± 0.10^a^	2.45 ± 0.14^a^	1.54 ± 0.09^a^	1.40 ± 0.08^a^
Monounsaturated fatty acid (MUFA)	22.36 ± 1.27^a^	22.92 ± 1.30^a^	19.35 ± 1.10^b^	11.73 ± 0.67^c^
Polyunsaturated fatty acid (PUFA)	41.94 ± 2.38^a^	47.85 ± 2.71^a^	41.15 ± 2.33^a^	46.40 ± 2.63^a^
PUFA/MUFA	1.88 ± 0.11^b^	2.09 ± 0.12^b^	2.13 ± 0.12^b^	3.96 ± 0.22^a^
Omega-3 (ω-3)	8.2 ± 0.46^b^	16.1 ± 0.91^a^	6.4 ± 0.36^c^	15.4 ± 0.87^a^
Omega-6 (ω-6)	33.74 ± 1.91^a^	31.75 ± 1.80^a^	34.80 ± 1.97^a^	31.00 ± 1.76^a^
Omega-6/Omega-3	4.11 ± 0.23^a^	1.97 ± 0.11^b^	5.48 ± 0.31^a^	2.01 ± 0.11^b^
Omega-7 (ω-7)	2.5 ± 0.14^a^	2.1 ± 0.12^a^	1.6 ± 0.09^b^	2.0 ± 0.11^a^
Omega-9 (ω-9)	19.86 ± 1.13^a^	20.80 ± 1.18^a^	17.73 ± 1.01^a^	9.76 ± 0.55^b^
PUFA/SFA (P/S)	1.18 ± 0.07^a^	1.66 ± 0.09^a^	1.05 ± 0.06^a^	1.12 ± 0.06^a^
MUFA/SFA (M/S)	0.63 ± 0.04^a^	0.79 ± 0.04^a^	0.49 ± 0.03^b^	0.28 ± 0.02^c^
Atherogenicity index (AI)	0.48 ± 0.03^a^	0.39 ± 0.02^a^	0.61 ± 0.03^a^	0.65 ± 0.04^a^
Thrombogenicity index (TI)	0.63 ± 0.04^a^	0.35 ± 0.02^c^	0.75 ± 0.04^a^	0.53 ± 0.03^b^
Hypocholesterolemic index (HI)	2.13 ± 0.12^a^	2.85 ± 0.16^a^	1.87 ± 0.11^a^	1.77 ± 0.10^a^
Peroxidizability index (PI)	50.70 ± 2.85^b^	64.88 ± 2.65^a^	47.98 ± 2.70^b^	62.09 ± 3.45^a^
Nutritive value index (NVI)	0.87 ± 0.05^b^	1.05 ± 0.06^a^	0.73 ± 0.04^c^	0.50 ± 0.03^d^

The values are expressed as means plus standard deviation of three replicates. Mean values with similar letters within a row are statistically similar, while mean values with different letters within a row are significantly different by the Tukey test (*P* < 0.05).

Generally, the major fatty acids in bitter orange, sweet orange, grapefruit, and mandarin peels were linoleic, palmitic, oleic, linolenic, stearic, palmitoleic, and myristic acids. Fatty acid composition from *Citrus maxima*^19^ and *C. natsudaidai* peel^20^ display comparable results to those in our study. Citrus peel oils have a low omega-6/omega-3 ratio, high omega-9 fatty acid (mainly oleic acid), high omega-6 fatty acids, and high omega-3 fatty acids (especial grapefruit and mandating peels) could be a better dietary source of fatty acids than most of the vegetable oils by authors opinion. Animals and humans cannot synthesize linoleic and linolenic acids, which are essential and provided exclusively from dietary sources. Citrus peel fatty acids could be a promising candidate for food supplements and ingredients to provide linoleic and linolenic acids^23^.

The medium-chain SFA (8–12 carbon atoms) is linked to lower blood pressure, better cardiac function, lower cancer risk, lower atherosclerosis risk, and lower low-density lipoprotein (LDL) cholesterol^24,25^. Moderate myristic acid consumption improves long-chain omega-3 fatty acid levels in plasma phospholipids, positively impacts cardiovascular health, and regulates key metabolic processes^26^. Palmitic acid (16:0, PA) is the most common saturated fatty acid found in the human body and can be provided in the diet or synthesized endogenously from other fatty acids. PA is a precursor for palmitoleic acid (16:1n-7, POA) synthesis by delta 9 desaturase. The disruption of PA homeostatic balance, implicated in different physiopathological conditions such as atherosclerosis, neurodegenerative diseases, and cancer, is often related to an uncontrolled PA endogenous biosynthesis, irrespective of its dietary contribution^27^. On the other hand, although it belongs to saturated fatty acids, stearic acid is not atherogenic and reduces cardiovascular and cancer risk^28^.

### Nutritional quality of citrus peel fatty acid

The results for the nutritional quality of fatty acids from bitter orange, sweet orange, grapefruit, and mandarin peels are presented in Table 2. The citrus peel contains PUFA, SFA, and MUFA, respectively. PUFA mainly contains omega-6 and omega-3, respectively. MUFA mainly contains omega-9 and omega-7, respectively. Considering the fatty acid nutritional quality, bitter orange, sweet orange, grapefruit, and mandarin are similar, although there are statistically significant differences in the total MUFA and omega-9 and omega-3 fatty acid content (P ≤ 5%). Grapefruit had the highest MUFA, omega-9, and omega-3 fatty acid. Mandarin had the lowest MUFA and omega-9 fatty acids (P ≤ 5%). Bitter orange and grapefruit are similar according to UFA/SFA, HI, UFA, PUFA/SFA, NVI, MUFA/SFA, MUFA, omega-9, PUFA, omega-3, PI, and omega-7. Sweet orange and mandarin are similar according to SFA, AI, TI, PUFA/MUFA, and omega-6/omega-3 (Table 2 and Fig. S3 in the supplemental file). Dietary variables are closely linked to the development of diet-related disease, and the consumption of fatty acids is thought to play a unique role. Because of their relevance to health, an adequate supply of UFA plays an important role in disease treatment. The ratio of SFA to UFA in biological membranes is an important feature. Reduced levels of UFA in membranes have been linked to various pathophysiological conditions, including cardiovascular disease, diabetes, and cancer^29^. MUFA are beneficial to biological membranes because they are liquid at body temperature but not quickly oxidized, allowing them to retain proper membrane fluidity. UFA also functions as a lipid hormone or lipokine, coordinating metabolic responses between tissues^30^. Accordingly, citrus peels fatty acids with a good balance of PUFA, SFA, MUFA, omega-6, omega-3, omega-9, and omega-7 are beneficial for human health and microalgae culture and biomass production.

### Biochemical of *Chlorella* supplemented with citrus peel

**Figure 2:**
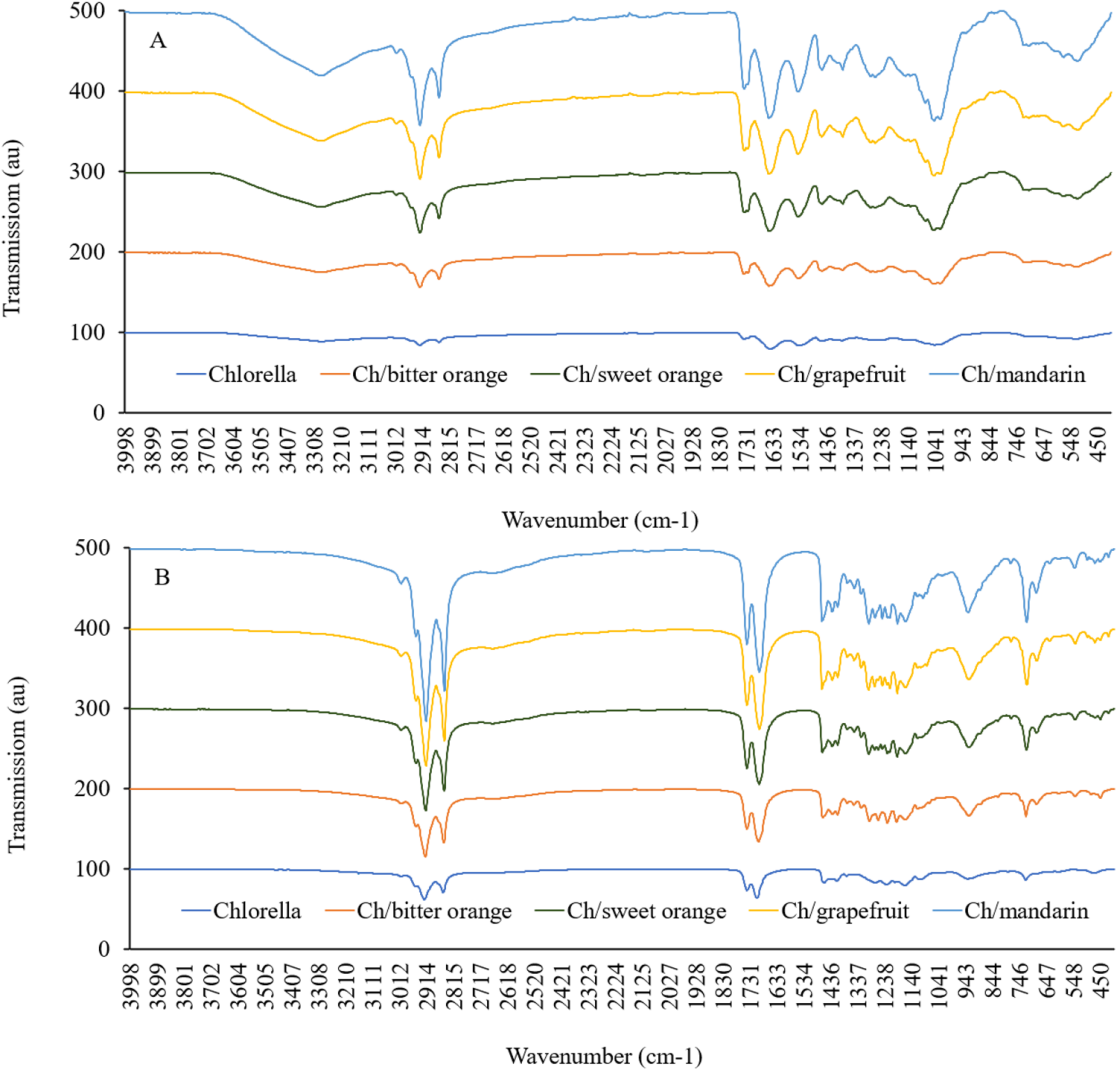
Fourier transform infrared spectrum of whole Chlorella (**A**) and Chlorella fatty acid (**B**) supplemented with fatty acid extract from bitter orange, sweet orange, grapefruit, and mandarin peels.

The FTIR patterns of *Chlorella* supplemented with fatty acid from bitter orange, sweet orange, grapefruit, and mandarin peels differ, indicating the various components in these products (Fig. 2). The 3700–3100 cm^−1^ bands are associated with the stretching vibrations of OH groups in water or aromatic molecules. Peaks in the 2900–2700 cm^−1^ region could be linked to the stretching vibration of lipid and fatty acid CH, CH2, and CH3 groups. The majority of the variations in FTIR patterns observed in the 2900–2700 cm^−1^ region can be attributed to a variation in fatty acid composition caused by citrus peel fatty acids. The C=C stretching of unsaturated fatty acids and the formation of MUFA and PUFA in *Chlorella* after supplementing with citrus peel can be related to the variations in FTIR patterns observed in the 1900–1500 cm^−1^ region. The stretching of C=O of amides, C=C of aromatics, N–H of amines, or carboxyl groups in proteins is represented by the bands in the region 1300–1000 cm^−1^. The bands at 1100–900 cm^−1^ are created by polysaccharide vibrations, including symmetric stretching of the C–O–C and OH groups. The vibration of terpenes and terpenoids causes the bands at 900–500 cm^−1^ ^31^.

The macronutrient composition of *Chlorella* and *Chlorella* supplemented with citrus peel fatty acids is stated in Table 3. Protein, carbohydrates, and lipids are the major components of *Chlorella*, respectively. *Chlorella* supplementation with citrus peel fatty acids increases total biomass and fat content while reducing carbohydrates and protein to some extent (P ≤ 5%). In this research, microalgae were separated by centrifugation, washed with distilled water, and then dried by lyophilization after culture. Wet biomass washing and lyophilization reduce the moisture content and salt (ash) of the dry matter of microalgae. *Chlorella/*bitter orange, *Chlorella*/grapefruit, *Chlorella*/sweet orange, and *Chlorella*/mandarin are similar according to energy, fat, moisture, and digestibility. *Chlorella* is different from other samples based on the ash, protein, dry matter, and carbohydrate (Table 3, Fig. S4 in supplemental file). Accordingly, supplementation with fatty acids from tested citrus peels modulates the macronutrient composition of *Chlorella*, especially fatty acids. Previous reports suggested that *Chlorella* biomass included an average of 40 g of protein, 18 g of carbohydrates, 12 g of fiber, and 10 g of lipids per 100 g of dry biomass^32–34^.

**Table 3:** Biochemical composition of Chlorella *vulgaris* supplemented with of bitter orange, sweet orange, grapefruit, and mandarin peel fatty acid.

Parameters	Chlorella	Chlorella + bitter orange	Chlorella + grapefruit	Chlorella + sweet orange	Chlorella + mandarin
Moisture (g/100 g fw)	6.75 ± 0.25^a^	7.37 ± 0.25^a^	6.86 ± 0.26^a^	7.57 ± 0.29^a^	7.45 ± 0.28^a^
Dry matter (g/100 g fw)	93.25 ± 3.51^a^	92.63 ± 3.51^a^	93.14 ± 3.51^a^	92.43 ± 3.48^a^	92.55 ± 3.49^a^
Ash (g/100 g dm)	7.85 ± 0.30^a^	6.54 ± 0.30^a^	6.65 ± 0.25^a^	6.85 ± 0.26^a^	6.63 ± 0.25^a^
Carbohydrate (g/100 g dm)	28.35 ± 1.07^a^	26.32 ± 1.07^a^	25.75 ± 0.97^a^	26.81 ± 1.01^a^	25.55 ± 0.96^a^
Protein (g/100 g dm)	38.85 ± 1.46^a^	33.50 ± 1.46^b^	32.75 ± 1.23^b^	34.29 ± 1.29^b^	34.22 ± 1.29^b^
Fat (g/100 g dm)	24.50 ± 0.92^b^	33.23 ± 0.92^a^	34.72 ± 1.31^a^	31.67 ± 1.19^a^	33.56 ± 1.26^a^
Energy (kcal/100 g)	489.30 ± 18.43^b^	538.35 ± 18.43^a^	546.49 ± 20.58^a^	529.43 ± 19.94^a^	541.12 ± 20.38^a^
Digestibility (g/100 g)	55.50 ± 2.09^a^	57.73 ± 2.09^a^	55.37 ± 2.09^a^	56.33 ± 2.12^a^	56.25 ± 2.12^a^

The values are expressed as means ± SD for three replicates experiments. Mean values with similar letters within a row are statistically similar, while mean values with different letters within a row are significantly different by the Tukey test (*P* < 0.05).

*fw* fresh weight, *Dm* dry matter.

### The fatty acid content of *Chlorella* supplemented with citrus peel

The FTIR patterns of fatty acid from *Chlorella* supplemented with bitter orange, sweet orange, grapefruit, and mandarin peel fatty acids are sated in Fig. 2. Peaks in the 2900–2700 cm^−1^ region could be linked to the stretching vibration of lipid and fatty acid CH, CH2, and CH3 groups. The majority of the variations in FTIR patterns observed in the 2900–2700 cm^−1^ region can be attributed to a variation in fatty acid composition caused by citrus peel fatty acids. The C=C stretching of unsaturated fatty acids and the formation of MUFA and PUFA in *Chlorella* after supplementing with citrus peel can be related to the variations in FTIR patterns observed in the 1900–1500 cm^−1^ region. The vibration of terpenes and terpenoids causes the bands at 1000–500 cm^−1^ ^31^. According to FTIR findings, *Chlorella* includes mostly proteins, carbohydrates, fatty acids, and lower hydrocarbons and terpenes. Because of the high level of essential fatty acid, high level of omega-3, and high levels of omega-6, microalgae such as *Chlorella* are preferred over bacteria and fungi as the single-cell oil for human eating. Carbohydrates (starch) and dietary fibers (cellulose) in microalgae biomass vary on average 20% and 12%, respectively. Because of the lack of lignin and hemicellulose, microalgae carbohydrates have been potential raw materials for bioethanol production. If the characteristics of *Chlorella* fatty acids are nutritionally attractive, *Chlorella* with citrus peel fatty acids can be marketed as a single-celled oil^33^.

Fatty acid composition of *Chlorella* and *Chlorella* supplemented with bitter orange, sweet orange, grapefruit, and mandarin peel fatty acids stated in Table 4. The primary fatty acids of *Chlorella* are palmitic, linoleic, oleic, alpha-linolenic, gamma-linolenic, hexadecatetraenoic, palmitoleic, hexadecadienoic, hexadecatrienoic, lauric and eicosapentaenoic acids (Table 4). To some extent, the fatty acid composition reported in *Chlorella* is similar to the previous results^32,34^. The fatty acid composition of *Chlorella* remains almost unchanged after supplementation with the fatty acids, as there are some statistically significant differences (Table 4). *Chlorella*/sweet orange, and *Chlorella*/mandarin according to docosahexaenoic acid, eicosanoic acid, heptadecanoic acid, myristic acid, tridecanoic acid, 4,7,10-hexadecatrienoic acid, pentadecanoic acid, 7,10,13-hexadecatrienoic acid, linoleic acid, palmitic acid, eicosapentaenoic acid, and lauric acid are correlated to each other (P ≤ 5%). *Chlorella*, *Chlorella*/bitter orange, and *Chlorella*/grapefruit samples are correlated according to 4,7,10,13-hexadecatetraenoic acid, gamma-linolenic acid, 6,9,12,15-octadecatetraenoic acid, oleic acid, palmitoleic acid, and alpha-linolenic acid (Table 4, Fig. S5 in supplemental file).

**Table 4:** Fatty acid composition (percent) and lipid nutritional quality of *Chlorella vulgaris* supplemented with of bitter orange, sweet orange, grapefruit, and mandarin peel fatty acid.

Fatty acid	Chlorella	Chlorella + bitter orange	Chlorella + grapefruit	Chlorella + sweet orange	Chlorella + mandarin
Decanoic acid (C10:0)	0.49 ± 0.03^b^	0.31 ± 0.02^b^	0.16 ± 0.01^c^	0.24 ± 0.01^c^	0.77 ± 0.05^a^
Dodecanoic acid (C12:0)	2.42 ± 0.14^b^	2.33 ± 0.14^b^	2.56 ± 0.15^b^	2.22 ± 0.13^b^	3.35 ± 0.20^a^
Tridecanoic acid (C13:0)	0.00 ± 0.00^b^	0.00 ± 0.00^b^	0.00 ± 0.00^b^	0.20 ± 0.01^b^	0.19 ± 0.01^a^
Tetradecanoic acid (C14:0)	0.44 ± 0.03^b^	0.52 ± 0.03^b^	0.56 ± 0.03^b^	0.88 ± 0.05^a^	1.02 ± 0.06^a^
Pentadecanoic acid (C15:0)	0.00 ± 0.00^b^	0.00 ± 0.00^b^	0.12 ± 0.01^a^	0.17 ± 0.01^a^	0.17 ± 0.01^a^
Hexadecanoic acid (C16:0)	23.16 ± 1.36^a^	23.96 ± 1.41^a^	22.08 ± 1.30^a^	24.13 ± 1.42^a^	24.94 ± 1.47^a^
9-Hexadecenoic acid (C16:1n7)	4.18 ± 0.25^a^	3.01 ± 0.18^a^	2.99 ± 0.18^a^	3.40 ± 0.20^a^	3.20 ± 0.19^a^
7,10-Hexadecadienoic acid (C16:2n6)	3.30 ± 0.19^a^	1.56 ± 0.09^b^	1.71 ± 0.10^b^	3.96 ± 0.23^a^	2.33 ± 0.14^b^
7,10,13-Hexadecatrienoic acid (C16:3n3)	2.45 ± 0.14^b^	1.23 ± 0.07^c^	1.40 ± 0.08^c^	6.54 ± 0.38^a^	6.57 ± 0.39^a^
4,7,10-Hexadecatrienoic acid (C16:3n6)	0.11 ± 0.01^b^	0.07 ± 0.00^b^	0.10 ± 0.01^b^	0.36 ± 0.02^a^	0.39 ± 0.02^a^
4,7,10,13-Hexadecatetraenoic acid (C16:4n3)	4.41 ± 0.26^a^	2.39 ± 0.14^b^	2.73 ± 0.16^b^	1.84 ± 0.11^b^	2.14 ± 0.13^b^
Heptadecanoic acid (C17:0)	0.00 ± 0.00^c^	0.22 ± 0.01^a^	0.14 ± 0.01^b^	0.24 ± 0.01^a^	0.35 ± 0.02^a^
Octadecanoic acid (C18:0)	1.69 ± 0.10^b^	2.78 ± 0.16^a^	2.15 ± 0.13^a^	1.93 ± 0.11^b^	2.18 ± 0.13^a^
9-Octadecenoic acid (C18:1n9)	17.80 ± 1.05^a^	19.14 ± 1.12^a^	18.52 ± 1.09^a^	12.53 ± 0.74^a^	10.20 ± 0.60^b^
9,12-Octadecadienoic acid (C18:2n6)	17.93 ± 1.05^b^	23.68 ± 1.39^a^	22.57 ± 1.33^a^	24.38 ± 1.43^a^	21.99 ± 1.29^a^
9,12,15-Octadecatrienoic acid (C18:3n3)	12.42 ± 0.73^b^	11.86 ± 0.70^b^	15.06 ± 0.89^a^	9.91 ± 0.58^c^	13.00 ± 0.76^b^
6,9,12-Octadecatrienoic acid (C18:3n6)	4.63 ± 0.27^a^	2.48 ± 0.15^b^	2.61 ± 0.15^b^	2.36 ± 0.14^b^	2.39 ± 0.14^b^
6,9,12,15-Octadecatetraenoic acid (C18:4n3)	1.95 ± 0.11^a^	0.98 ± 0.06^b^	1.20 ± 0.07^a^	0.99 ± 0.06^b^	1.06 ± 0.06^b^
Eicosanoic acid (C20:0)	0.00 ± 0.00^c^	0.44 ± 0.03^a^	0.25 ± 0.01^b^	0.52 ± 0.03^a^	0.59 ± 0.03^a^
Arachidonic acid (C20:4n6)	0.00 ± 0.00^b^	0.00 ± 0.00^b^	0.00 ± 0.00^b^	0.00 ± 0.00^b^	0.00 ± 0.00^b^
5,8,11,14,17-Eicosapentaenoic acid (C20:5n3)	2.00 ± 0.12^a^	2.41 ± 0.14^a^	2.40 ± 0.14^a^	2.47 ± 0.15^a^	2.27 ± 0.13^a^
13,16-Docosadienoic acid (C22:2n6)	0.00 ± 0.00^c^	0.18 ± 0.01^b^	0.12 ± 0.01^b^	0.30 ± 0.02^a^	0.31 ± 0.02^a^
Docosahexaenoic acid (C22:2n6)	0.00 ± 0.00^a^	0.00 ± 0.00^a^	0.00 ± 0.00^a^	0.00 ± 0.00^a^	0.00 ± 0.00^a^
Total	99.37 ± 5.84^a^	99.55 ± 5.85^a^	99.45 ± 5.84^a^	99.55 ± 5.85^a^	99.42 ± 5.84^a^
Saturated fatty acid (SFA)	28.21 ± 1.66^b^	30.56 ± 1.80^a^	28.04 ± 1.65^b^	30.52 ± 1.79^a^	33.57 ± 1.97^a^
Unsaturated fatty acid (UFA)	71.16 ± 4.18^a^	68.99 ± 4.05^a^	71.41 ± 4.20^a^	69.03 ± 4.06^a^	65.85 ± 3.87^ab^
UFA/SFA	2.52 ± 0.15^a^	2.26 ± 0.13^a^	2.55 ± 0.15^a^	2.26 ± 0.13^a^	1.96 ± 0.12^a^
Monounsaturated fatty acid (MUFA)	21.98 ± 1.29^a^	22.15 ± 1.30^a^	21.51 ± 1.26^a^	15.92 ± 0.94^b^	13.40 ± 0.79^b^
Polyunsaturated fatty acid (PUFA)	49.19 ± 2.89^a^	46.84 ± 2.75^a^	49.90 ± 2.93^a^	53.10 ± 3.12^a^	52.45 ± 3.08^a^
PUFA/MUFA	2.24 ± 0.13^a^	2.11 ± 0.12^a^	2.32 ± 0.14^a^	3.33 ± 0.20^a^	3.91 ± 0.23^a^
Omega-3 (ω-3)	23.2 ± 1.36^a^	18.9 ± 1.11^c^	22.8 ± 1.34^ab^	21.7 ± 1.28^ab^	25.0 ± 1.47^a^
Omega-6 (ω-6)	25.97 ± 1.53^a^	27.97 ± 1.64^a^	27.11 ± 1.59^a^	31.36 ± 1.84^a^	27.41 ± 1.61^a^
Omega-6/Omega-3 (ω-6/ω-3)	1.12 ± 0.07^a^	1.48 ± 0.09^a^	1.19 ± 0.07^a^	1.44 ± 0.08^a^	1.09 ± 0.06^a^
Omega-7 (ω-7)	4.2 ± 0.25^a^	3.0 ± 0.18^a^	3.0 ± 0.18^a^	3.4 ± 0.20^a^	3.2 ± 0.19^a^
Omega-9 (ω-9)	17.80 ± 1.05^a^	19.14 ± 1.12^a^	18.52 ± 1.09^a^	12.53 ± 0.74^b^	10.20 ± 0.60^b^
PUFA/SFA (P/S)	1.74 ± 0.10^a^	1.53 ± 0.09^a^	1.78 ± 0.10^a^	1.74 ± 0.10^a^	1.56 ± 0.09^a^
MUFA/SFA (M/S)	0.78 ± 0.05^a^	0.72 ± 0.04^a^	0.77 ± 0.05^a^	0.52 ± 0.03^a^	0.40 ± 0.02^a^
Atherogenicity index (AI)	0.38 ± 0.02^a^	0.41 ± 0.02^a^	0.38 ± 0.02^a^	0.43 ± 0.03^a^	0.49 ± 0.03^a^
Thrombogenicity index (TI)	0.27 ± 0.02^a^	0.33 ± 0.02^a^	0.27 ± 0.02^a^	0.30 ± 0.02^a^	0.29 ± 0.02^a^
Hypocholesterolemic index (HI)	2.04 ± 0.12^a^	2.23 ± 0.13^a^	2.48 ± 0.15^a^	1.87 ± 0.11^a^	1.74 ± 0.10^a^
Peroxidizability index (PI)	98.40 ± 5.78^a^	85.20 ± 5.01^a^	93.40 ± 5.49^a^	93.51 ± 5.50^a^	96.09 ± 5.65^a^
Nutritive value index (NVI)	0.84 ± 0.05^a^	0.92 ± 0.05^a^	0.94 ± 0.06^a^	0.60 ± 0.04^a^	0.50 ± 0.03^a^

The values are expressed as means plus standard deviation of three replicates. Mean values with similar letters within a row are statistically similar, while mean values with different letters within a row are significantly different by the Tukey test (*P* < 0.05).

### Fatty acid nutritional quality of *Chlorella* supplemented with citrus peel

Fatty acid nutritional quality of *Chlorella* and *Chlorella* supplemented with bitter orange, sweet orange, grapefruit, and mandarin peel fatty acids stated in Table 4. *Chlorella* manly contains PUFA, SFA, and MUFA, respectively. PUFA mainly contains omega-6 and omega-3, respectively. MUFA contains omega-9 and omega-7, respectively (Table 4). *Chlorella* had nutritionally acceptable AI, TI, omega-6/omega-3, HI, PI, and NVI (Table 4). Supplementation of *Chlorella* with citrus peel fatty acids increases the total biomass and lipid content, while carbohydrate and protein, to some extent, decrease (P ≤ 5%). *Chlorella* and supplemented *Chlorella*'s nutritional quality and fatty acid composition are similar, but total fatty acid increased. *Chlorella*, *Chlorella/*bitter orange, and *Chlorella*/grapefruit are correlated according to MUFA/SFA, MUFA, omega-9, NVI, UFA, UFA/SFA, HI, and PUFA/SFA. *Chlorella*/sweet orange and *Chlorella*/mandarin are similar according to PUFA/MUFA, AI, SFA, PUFA, omega-3, and omega-6 (Table 4, Fig. S6 in supplemental file).

Balanced fatty acid in the *Chlorella* leads to an acceptable AI, TI, HI, PUFA/SFA, omega-6/omega-3 ratio, PI, and NVI, making *Chlorella* suitable for food supplements. Because the ratio of UFA/SFA in the *Chlorella* is above 1.5, it can recommend a promising candidate for raising high-density lipoprotein (HDL) cholesterol and depressing LDL cholesterol. Lowering triglycerides, preventing inflammation, increasing mitochondrial biogenesis, restoring insulin sensitivity, reducing central body fat, and suppressing thrombosis and inflammation are benefits of an omega-3-rich diet^35^. Increased blood viscosity, vasoconstriction, and promoting prothrombotic, proinflammatory, and proaggregatory factors are linked to a diet with high omega-6 fatty acids. Maintaining good health necessitates a sensible omega-6/omega-3 ratio^36^. *Chlorella* supplemented with citrus peel is beneficial as a rich source of essential fatty acids and the excellent omega-6/omega-3 ratio, which is crucial in dietary supplements to prevent and manage chronic illnesses and obesity problems^34^.

A PUFA/SFA ratio of over 0.45 is advised to avoid cardiovascular disease and several chronic illnesses in human diets, including cancer. The recommended PUFA/SFA ratio, which indicates the quality of lipid nutrition in a given diet, is 1–2, decreasing blood cholesterol and lowering the risk of heart disease^34^. Low AI and TI values indicate that foodstuffs have a greater preventive impact in avoiding heart and coronary illnesses, decreasing overall and abdominal obesity, and reducing diabetes in pregnant women. High PUFA levels are closely connected to high HI fatty acid ratios, which are more favorable for human health since they are regarded as the optimal quantity of cholesterol. The PI is a measure of PUFA sensitivity to oxidation. The range of 70–90 is a favorable PI ratio representing the lipid nutritional quality of a certain meal and lowers blood cholesterol and cardiovascular disease. A higher amount of fatty acid oxidation is associated with higher PI levels. However, high PI levels due to high omega-3 PUFA and omega-6 PUFA lead to greater antioxidant and anti-inflammatory actions^36^.

Citrus peel oil produces a rise in the nutritional quality of *Chlorella* due to the findings, which is a valuable gain for *Chlorella* applications such as food supplements, medicinal benefits, and biodiesel generation. Citrus peel oil can be converted to acetyl-CoA, then converted to palmitic acid, and subsequently to an unsaturated fatty acid by elongases and desaturases. (1) Fatty acids may be incorporated into the phospholipids and glycolipids of the plasma membrane. (2) Fatty acids may be incorporated into the triglyceride and used as a storage resource. (3) Fatty acids may be incorporated into the metabolic pathway of fatty acids and converted to omega-3 and omega-6 fatty acids. Citrus peel oil enters directly into the synthesis pathway of unsaturated fatty acids like linoleic acid and linolenic acid^35^. In the omega-3 pathway, 15-desaturase converts linoleic acid to alpha-linolenic acid. In the omega-6 pathway, 6-desaturase transforms linoleic acid into gamma-linolenic acid^24^. When the diet contains high linoleic acid (citrus peel oil) levels, placed in the *Chlorella* culture medium, ∆15-desaturase is involved in the biosynthesis of alpha-linolenic acid from linoleic acid^11^. In contrast, ∆6-desaturase is involved in the biosynthesis of gamma-linolenic acid from linoleic acid^11^. Adding citrus peel oil to the culture medium of *Chlorella* leads to an acceptable production of omega-9, omega-6, omega-3, and nutritionally suitable omega-6/omega-3 ratio^26^. Accordingly, humans whose diet is rich in these fatty acids have lower inflammation and better insulin sensitivity than those with saturated fatty acids in their diet. So, these diets are beneficial for human health^26^. The use of fatty acids by microalgae is complex, and explanation in this case is very difficult and need more studies.

## Conclusion

Citrus peel oil is rich in carbohydrates, proteins, and lipids. Given fatty acid content, citrus peels are rich sours of linoleic acid, palmitic acid, oleic acid, linolenic acid, stearic acid, polyunsaturated fatty acid, saturated fatty acids, and monounsaturated fatty acids with good nutritional quality. With this nutritional quality, citrus peels could be used as an inexpensive nutrient for microalgae growth to improve microalgae biomass. But the direct use of citrus peel has several limitations due to antinutrient materials in the citrus peels. Partial extraction of fatty acids and removing antinutrients could reduce these limitations. Supplementing *Chlorella* with fatty acids in citrus peel increases total biomass and lipid content. Although further research is needed to increase the lipid and fatty acid content of *Chlorella*, recent findings suggest that citrus peel may be utilized to grow microalgae and provide microalgal biomass for nutritional supplements at a low cost.

## Supplementary Information

Supplementary Figures.

## Data Availability

The data that support the findings of this study are available from the corresponding author, upon reasonable request.
