# Supplementary material for: Manipulation of fatty acid profile and nutritional quality of Chlorella vulgaris by supplementing with citrus peel fatty acid
Source: Sci Rep. 2022 May 17;12:8151. doi: 10.1038/s41598-022-12309-y (PMC9114136; doi:10.1038/s41598-022-12309-y)
Supplement: Supplementary file 1 — Supplementary Figures. [file 41598_2022_12309_MOESM1_ESM.doc]

**Manipulation of fatty acid profile and nutritional quality of *Chlorella vulgaris* by supplementing with citrus peel fatty acid**

Kourosh Ghodrat Jahromi^1^, Zhila Heydari Koochi^1^, Gholamreza Kavoosi^1^ *, Alireza Shahsavar^2^

1. School of Agriculture, Department of Biotechnology, Shiraz University, Shiraz, Islamic Republic of Iran.

2. Department of Horticultural Science, School of Agriculture, Shiraz University, Shiraz, Iran

* Corresponding author: ghkavoosi@shirazu.ac.ir


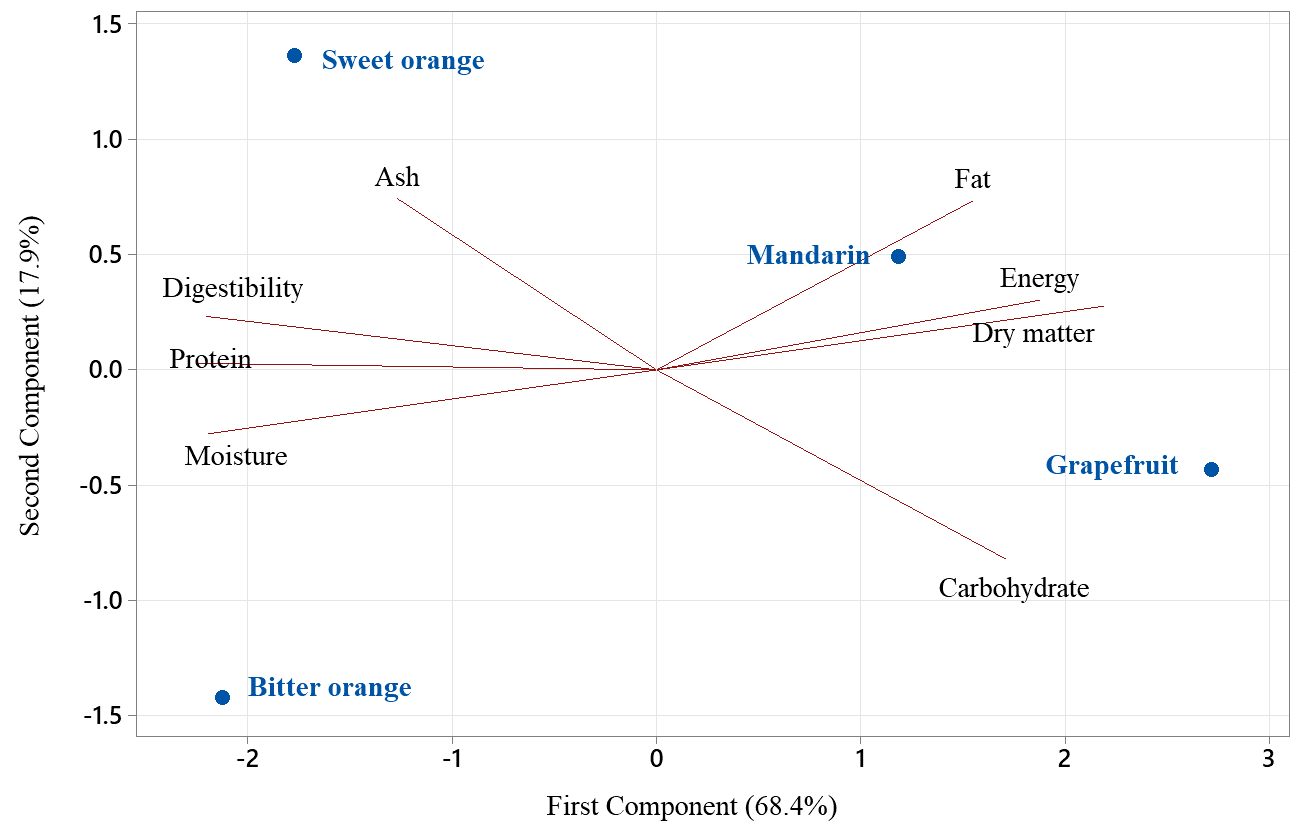
Figure S1. Principal Component Analysis (PCA) biplot illustrating the relationships among the biochemical composition of bitter orange, sweet orange, grapefruit, and mandarin. The first principal components (PC1) and second principal components (PC2) accounted for 86.3% of the overall variation in the changes. The PC1 accounting for 68.4% (eigen value=5.47), and the PC2 accounting for 17.9% (eigen value=1.43). The PC1 at positive side is correlated with bitter orange and grapefruit and dry matter (0.400), energy (0.343), carbohydrate (0.312), and fat (0.283). PC1 at negative side is correlated with sweet orange and mandarin and protein (-0.411), and digestibility (-0.403), moisture (-0.400), and ash (-0.232). The PC2 at positive side is correlated with sweet orange and bitter orange and ash (0.518), fat (0.511), energy (0.211), dry matter (0.193), and digestibility (0.162). PC2 at negative side is correlated with mandarin and grapefruit, and carbohydrate (-0.571), and moisture (-0.193).


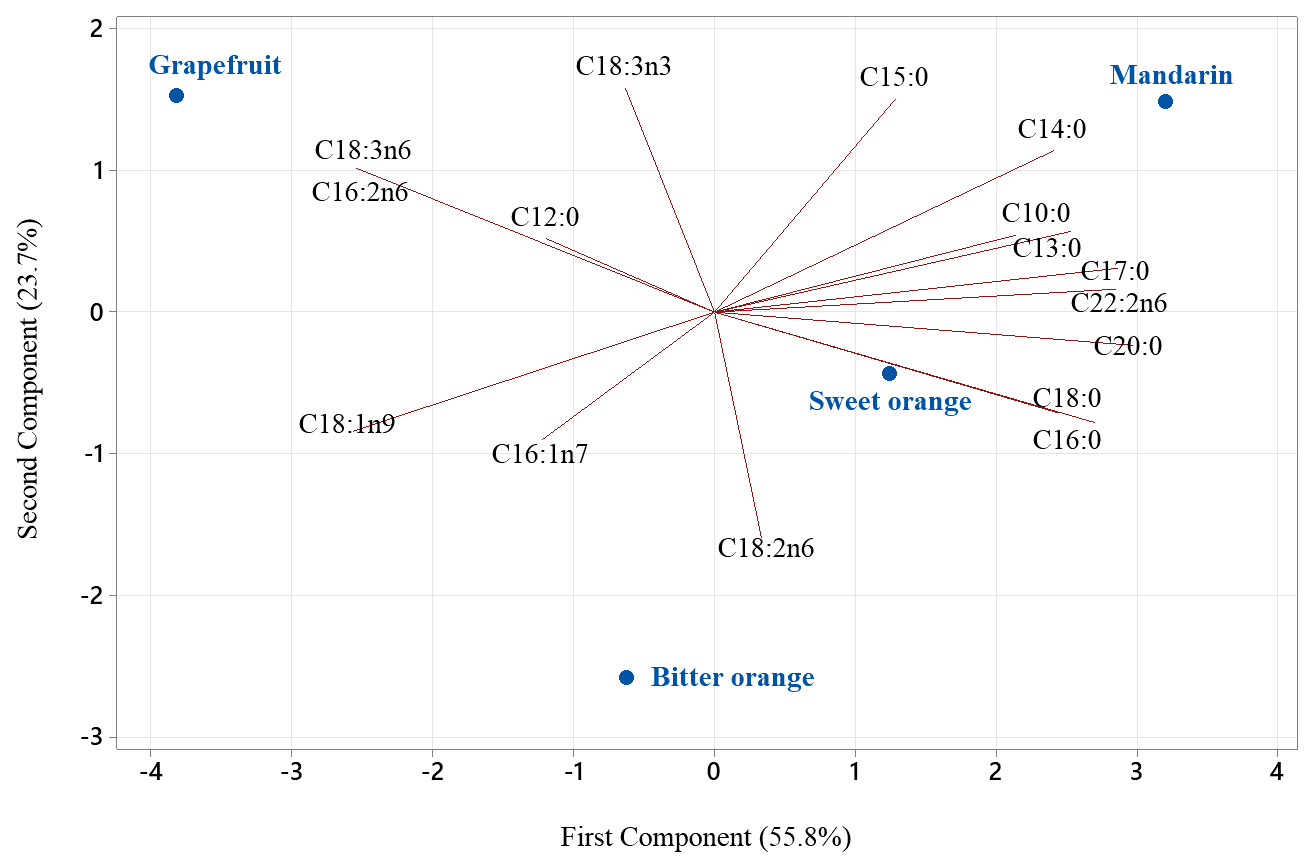


Figure S2. Principal Component Analysis (PCA) biplot illustrating the relationships among the fatty acid composition of bitter orange, sweet orange, grapefruit, and mandarin. The PC1 and PC2 accounted for 79.5% total variance in fatty acid composition. PC1 accounting for 55.8% (eigen value=8.92) and the PC2 for 23.7% (eigen value=3.79). The PC1 at positive side is correlated with sweet orange, and mandarin, and C20:0 (0.332), C17:0 (0.320), C22:2n6 (0.319), C16:0 (0.302), C13:0 (0.283), C18:0 (0.274), C14:0 (0.270), C10:0 (0.239), and C15:0 (0.144) variables. The PC1 at negative side is correlated with bitter orange, grapefruit, and C18:1n9 (-0.287), C16:2n6 (-0.285), and C18:3n6 (-0.285), C16:1n7 (-0.137), and C12:0 (-0.134) variables. The PC2 at positive side is correlated with grapefruit, and mandarin, and C18:3n3 (0.417), C15:0 (0.397), C14:0 (0.301), C16:2n6 (0.269), C18:3n6 (0.269), C13:0 (0.150), C10:0 (0.143), and C12:0 (0.137) variables. PC2 at negative side is correlated with bitter orange, and sweet orange, and C18:2n6 (-0.419), C16:1n7 (-0.238), C18:1n9 (-0.221), C16:0 (-0.206), and C18:0 (-0.188) variables.


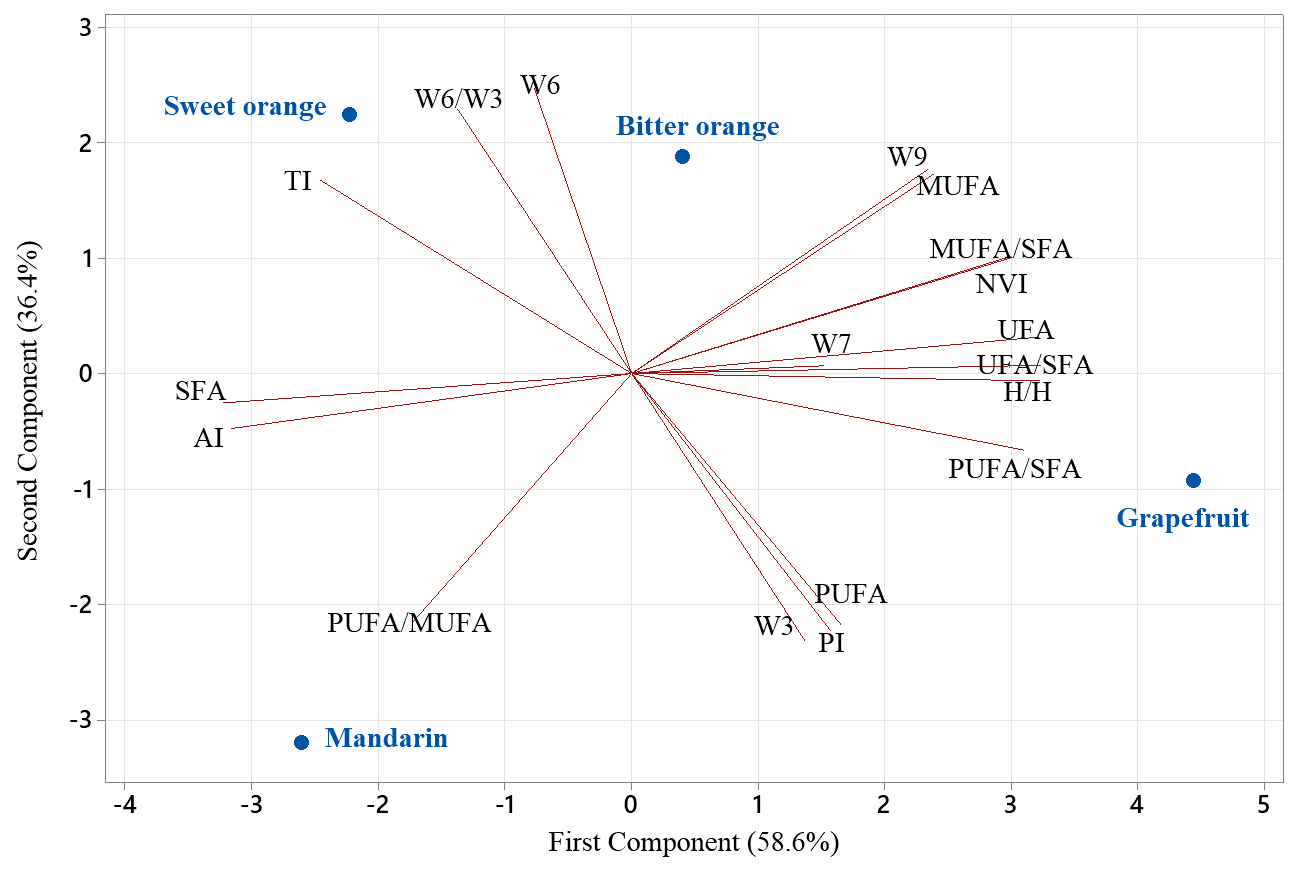


Figure S3. Principal Component Analysis (PCA) biplot illustrating the relationships among the fatty acid nutritional quality of bitter orange, sweet orange, grapefruit, and mandarin. PC1 and PC2 were responsible for 94.9% of the overall variation in nutritional quality. The PC1 accounting for 58.6% (eigen value=10.54) and the PC2 for 36.4% (eigen value=6.54). The PC1 at positive side is correlated with bitter orange, grapefruit, and UFA/SFA (0.306), H/H (0.306), UFA (0.305), PUFA/SFA (0.294), NVI (0.283), MUFA/SFA (0.283), MUFA (0.227), ω-9 (0.222), PUFA (0.157), ω-3 (0.130), PI (0.149), and ω-7 (0.144) variables. The PC1 at negative side is correlated with sweet orange and mandarin and SFA (-0.306), AI (-0.300), TI (-0.233), PUFA/MUFA (-0.165), and ω-6/ω-3 (-0.130) variables. The PC2 at positive side is correlated with bitter orange, and sweet orange, and ω-6 (0.377), ω-6/ω-3 (0.350), ω-9 (0.270), MUFA (0.264), NVI (0.154), and MUFA/SFA (0.153) variables. PC2 at negative side is correlated with grapefruit and mandarin and ω-3 (-0.354), PI (-0.340), PUFA (-0.333), PUFA/MUFA (-0.330), and PUFA/SFA (-0.101) variables.


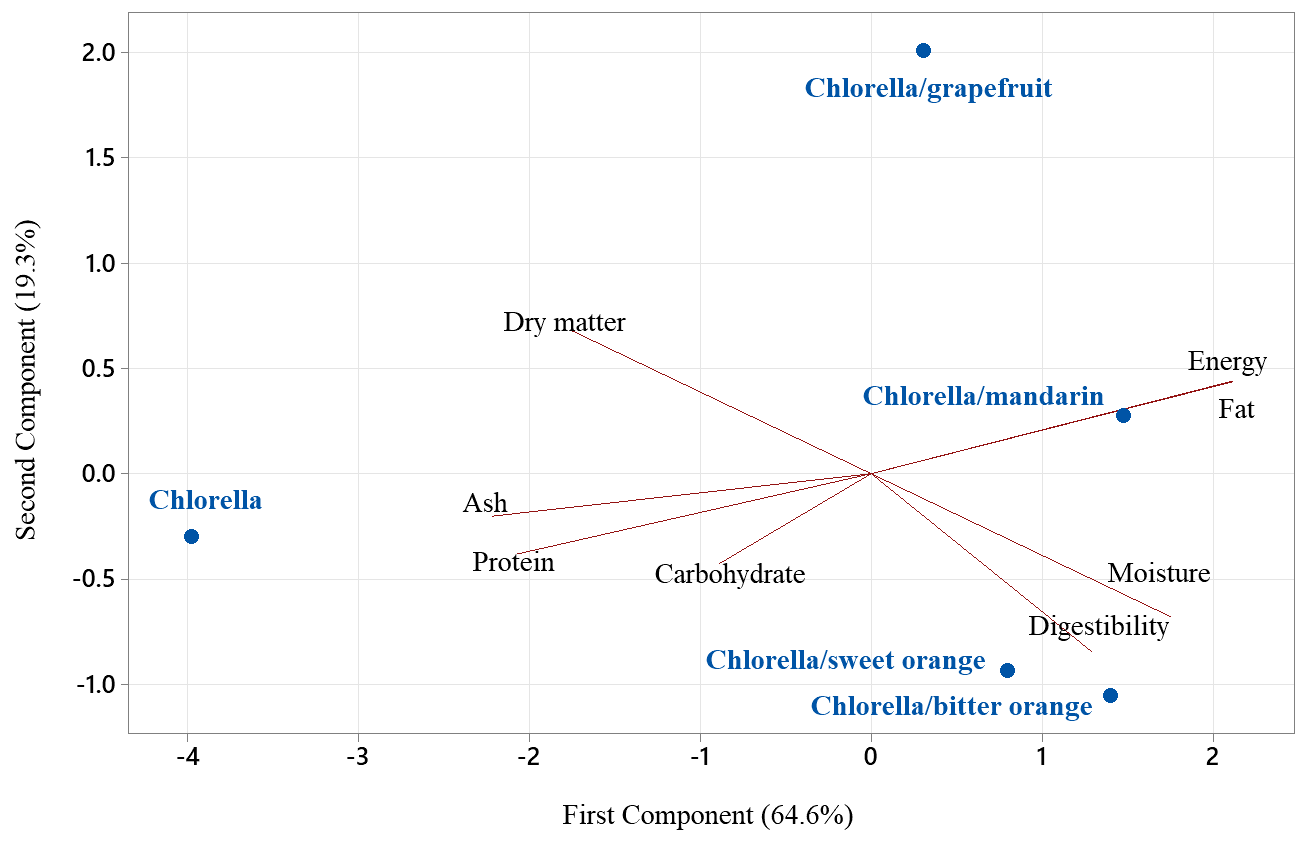
Figure S4. Principal Component Analysis (PCA) biplot illustrating the relationships among the biochemical composition of Chlorella supplemented with bitter orange, sweet orange, grapefruit, and mandarin. The biochemical composition of Chlorella and Chlorella supplemented with citrus peel fatty acids is stated in Table 3. PC1 and PC2 were responsible for 83.9% of the overall variation in Chlorella chemical composition. The PC1 accounting for 64.6% (eigen value=5.16) and the PC2 for 19.3% (eigen value=1.54). The PC1 at positive side is correlated with Chlorella/bitter orange, Chlorella/grapefruit, Chlorella.sweet orange, and Chlorella/mandarin, and energy (0.409), fat (0.408), moisture (0.339), and digestibility (0.250). PC1 at negative is correlated with Chlorella and ash (-0.429), protein (-0.401), dry matter (-0.339), and carbohydrate (-0.172). The PC2 at positive side is correlated with Chlorella/graperfruit, and Chlorella/mandarin and dry matter (0.439), fat (0.284), and energy (0.283). PC2 at negative side is correlated with Chlorella, Chlorella/bitter orange, and Chlorella/sweet orange and digestibility (-0.547), moisture (-0.439), carbohydrate (-0.276), protein (-0.247), and ash (-0.131)


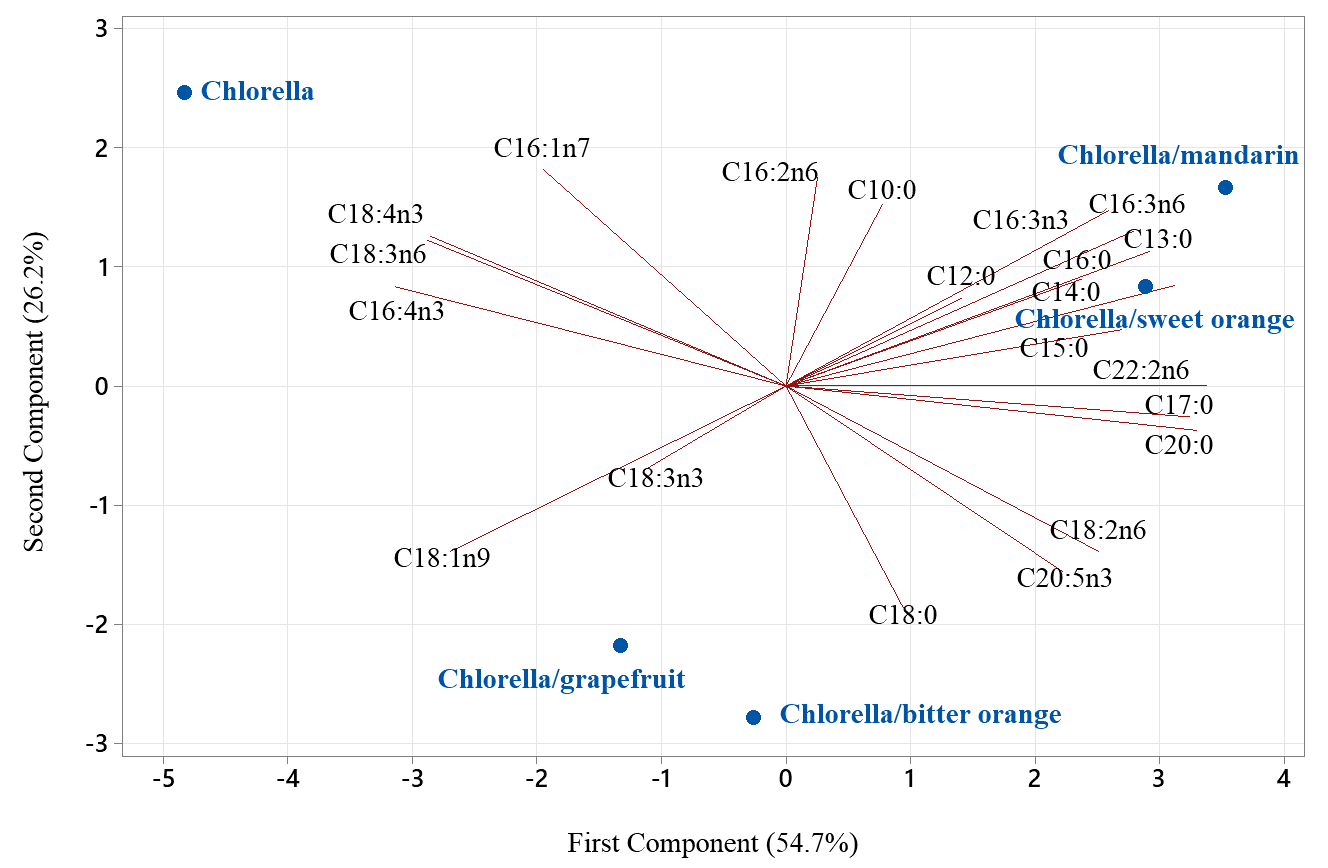


Figure S5. Principal Component Analysis (PCA) biplot illustrating the relationships among the fatty acid composition of Chlorella supplemented with bitter orange, sweet orange, grapefruit, and mandarin. The PC1 and PC2 accounted for 81.0% total variation in fatty acid of supplemented Chlorella. The PC1 accounting for 54.7% (eigen value=11.49) and the PC2 for 26.2% (eigen value=5.51). The PC1 at positive side is correlated with Chlorella/sweet orange, and Chlorella/mandarin, and C22:2n6 (0.294), C20:0 (0.287), C17:0 (0.282), C14:0 (0.271), C13:0 (0.254), C16:3n6 (0.243), C15:0 (0.235), C16:3n3 (0.226), C18:2n6 (0.219), C16:0 (0.207), C20:5n3 (0.194), and C12:0 (0.123). PC1 at negative side is correlated with Chlorella, Chlorella/bitter orange, and Chlorella/grapefruit and C16:4n3 (-0.273), C18:3n6 (-0.250), C18:4n3 (-0.248) C18:1n9 (-0.235), C16:1n7 (-0.170), C18:3n3 (-0.100). The PC2 at positive is correlated with Chlorella, Chlorella/sweet orange, and Chlorella/mndarin and C16:1n7 (0.331), C16:2n6 (0.317), C10:0 (0.276), C16:3n3 (0.268), C16:3n6 (0.236), C18:4n3 (0.228), C18:3n6 (0.222), C13:0 (0.205), C16:0 (0.163), C14:0 (0.153), C16:4n3 (0.151), and C12:0 (0.134). PC2 at negative side is correlated with Chlorella/bitter orange, and Chlorella /grapefruit and C18:0 (-0.338), C20:5n3 (-0.283), C18:1n9 (-0.253), C18:2n6 (-0.252), and C18:3n3 (-0.129).


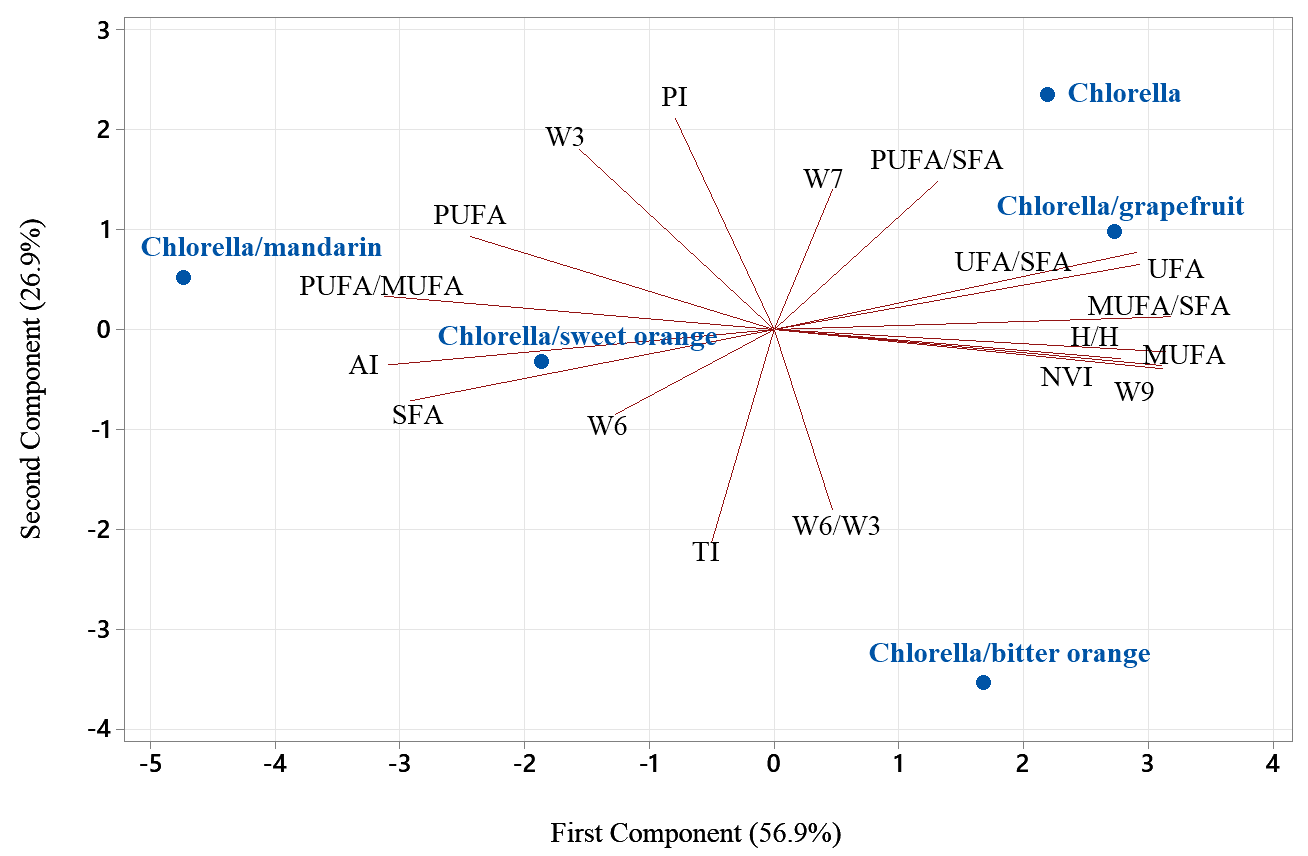


Figure S6. Principal Component Analysis (PCA) biplot illustrating the relationships among the fatty acid nutritional quality of Chlorella supplemented with bitter orange, sweet orange, grapefruit, and mandarin. The PC1 and PC2 accounted for 83.8% total variance in nutritional quality. The PC1 accounted for 56.9% (eigen value=10.24) and the PC2 for 26.9% (eigen value=4.84). The PC1 at positive side is correlated with Chlorella, Chlorella/bitter orange, and Chlorella/grapefruit, and MUFA/SFA (0.311), MUFA (0.306), ω-9 (0.304), NVI (0.303), UFA (0.286), UFA/SFA (0.284), H/H (0.271), and PUFA/SFA (0.128). PC1 at negative side is correlated with Chlorella/sweet orange, and Chlorella/mandarin, and PUFA/MUFA (-0.305), AI (-0.302), SFA (-0.285), PUFA (-0.238), ω-3 (-0.152), and ω-6 (-0.125). The PC2 at positive side is correlated with Chlorella, Chlorella/mandarin, and Chlorella/grapefruit and PI (0.436), ω-3 (0.372), PUFA/SFA (0.307), ω-7 (0.290), PUFA (0.192), UFA/SFA (0.159), and UFA (0.135). PC2 at negative side is correlated with Chlorella/bitter orange, and Chlorella/sweet orange and TI (-0.438), ω-6/ω-3 (-0.373), ω-6 (-0.176), and SFA (-0.147).
